# Psychological Capital and Family Satisfaction among Employees: Do Occupational Stressors Moderate the Relationship?

**DOI:** 10.3390/ijerph182212260

**Published:** 2021-11-22

**Authors:** Oi-Ling Siu, Qianting Kong, Ting-Kin Ng

**Affiliations:** Department of Applied Psychology, Lingnan University, 8 Castle Peak Road, Tuen Mun, New Territories, Hong Kong, China; Acc.su.2021@gmail.com (Q.K.); ngtingkin@gmail.com (T.-K.N.)

**Keywords:** occupational stress, family, positive psychology

## Abstract

The COVID-19 pandemic has created more occupational stressors, particularly work–family interface issues. The purpose of this study was to investigate the moderating role of occupational stressors in the relationship between a personal resource (psychological capital) and family satisfaction. A cross-sectional study was carried out with a sample of 787 employees (367 males, 420 females) from the Greater Bay Area of China between October and November 2020. Participants completed an online survey which included the Chinese version of the Psychological Capital Questionnaire, measures of occupational stressors from the Work Stress Management DIY Kit and a measure of family satisfaction. Latent moderated structural equation modeling revealed that family satisfaction was positively associated with psychological capital and negatively associated with occupational stressors. Furthermore, occupational stressors weakened the positive association between psychological capital and family satisfaction. These findings provided empirical evidence for the work–home resources model and may suggest that it would be beneficial to boost psychological capital and reduce occupational stressors of employees.

## 1. Introduction

The COVID-19 pandemic has sped up changes in work life, especially among those working from home, which has become a trend worldwide. Shutdowns and stay-at-home policies have led to lessened personal face-to-face contact and created more work–family interface issues, particularly for those who have children and elders at home. Family satisfaction is relatively under-explored as an outcome of occupational stressors. Work and family domains are two of the most central domains of adult working life. Apart from testing the impacts of various factors on outcomes within the same domain [1,2,3], a considerable amount of research has been carried out to explore the interface between the work and family domains [4,5,6]. Some scholars have argued that work and family roles can interfere with one another (work–home conflict) [7,8]. On the contrary, other scholars have contended that work and family roles may enrich each other (work–home enrichment) [9,10]. The work–home resources model [11] integrates the processes of work–home conflict and work–home enrichment by considering the demands and resources in one domain as the causes of outcomes in the other domain.

Family satisfaction refers to the degree to which individuals are satisfied with their life in the family domain [12,13,14]. A high level of family satisfaction is beneficial for individuals. For example, spina bifida patients with increased family satisfaction experience higher quality of life in physical and psychological domains [15]. A higher level of family satisfaction is predictive of lower negative effects, better mental health [16], higher life satisfaction [17,18] and greater longevity [19]. A large amount of work has therefore been conducted to investigate the predictors of family satisfaction. Research has shown that family satisfaction can be predicted by factors in the family domain, including participation in family routines with family members [20], family support [3], family communication [21] and so on. Other findings have revealed that support from co-workers can predict family satisfaction [22], and that individuals with team resources at the workplace are more likely to feel satisfied with their family life [23], suggesting that family satisfaction is predicted not only by antecedents in the family domain but also by factors in the work domain [24].

Psychological capital is a higher-order positive psychological construct defined as a set of personal resources that can mobilize other resources, such as cognitive skills [11,25,26]. Psychological capital is comprised of four components: self-efficacy, optimism, hope and resiliency [25,27]. Self-efficacy refers to confidence about accomplishing particular tasks. Optimism refers to positive expectations for succeeding in the present and future. Hope refers to perseverance with achieving one’s goals and consideration of alternative strategies to attain these goals when obstacles occur. Resiliency refers to the capacity to bounce back from adversity or difficulties [25]. Research has shown that individuals with higher psychological capital at work are more satisfied with their job, perform better at work, experience fewer work stresses and have lower turnover intention [27,28]. This suggests that psychological capital at work has a positive effect on organizational outcomes. Recent research suggested that the positive effect of psychological capital remains during the pandemic [29,30].

According to the expansionist approach of the role theory, a role may generate energy that can be used for performance in this role as well as in other roles [31]. Greenhaus and Powell [32] further postulated a work–home enrichment model, which suggests that a range of resources generated in a role, including psychological resources, can benefit another role. Greenhaus and Powell [32] also proposed two paths for work–home enrichment. First, in the instrumental path, resources generated in one role can be directly used in another role. Second, in the effective path, resources generated in one role can improve the positive effect in this role, which in turn can enhance performance in another role. According to the work–family enrichment model, psychological capital may mobilize other personal resources such as cognitive skills at work, which may, in turn, be transferred to the family domain and lead to an improvement in family satisfaction. Sava et al. [33] found that psychological capital contributed to improved self-regulation, suggesting that psychological capital could mobilize cognitive skills that could be utilized in the family domain. On the other hand, a high level of psychological capital may enable individuals to marshal other resources to perform well at work, and therefore promote their positive effects, which, in turn, may improve their performance in the family domain and hence increase family satisfaction.

Consistent with the work–home resources model [11], work–home enrichment occurs when resources at work increase personal resources, which can be employed in the family domain. On the other hand, work–home conflict arises when resources are depleted by demands at work. Occupational stressors are an example of those demands in the work domain.

In recent decades, ample attention has been paid to occupational stressors. Researchers have identified the main occupational stressors in different societies. It has been postulated that job insecurity, interpersonal conflicts, quantitative workload, organizational constraints and work–home interface are the most common occupational stressors among employees in Hong Kong [34]. Occupational stressors are harmful to employees. In the organizational domain, high occupational stressors are associated with decreased job satisfaction [35], low work productivity [36], frequent presenteeism [34] and increased turnover rate [37]. The work–home resources model suggests that demands in the work domain could have negative effects on outcomes in the home domain through draining personal resources [11]. Research has shown that high organizational politics was negatively associated with satisfaction of family life [38]. Findings from a qualitative study also suggested that occupational stressors may cause family conflict, decreased family unity and low family performance [39]. Accordingly, when employees experience high levels of occupational stressors, their personal resources would diminish, resulting in decreased involvement in the family domain and, therefore, in low family satisfaction.

Additionally, occupational stressors may moderate the association between psychological capital and family satisfaction. Although it is suggested that psychological capital could mobilize resources for task completion [11,26,33], occupational stressors may interfere with this process. Findings in the field of neuroscience revealed that stress could impair the function of the prefrontal cortex, which plays a key role in cognitive abilities [40,41]. Behavioral experiments also found that induced stress would compromise cognitive functions [42]. These findings may suggest that stress may impede the mobilization of cognitive resources. Thus, when levels of occupational stressors are low, psychological capital may be able to effectively deploy cognitive resources to complete tasks at work, which could generate a positive effect. This positive effect, as well as remaining cognitive resources, improves performance in the family domain, which may also lead to increased family satisfaction. However, when occupational stressors are high, fewer cognitive resources may be mobilized by psychological capital for tasks at work, so positive effects and cognitive resources that could be utilized for the family role may decrease, which may result in lower levels of family satisfaction. In other words, the positive association between psychological capital and family satisfaction may be weaker in situations where occupational stressors are high than where occupational stressors are low.

The present study aims to examine the roles of psychological capital and occupational stressors in family satisfaction. We hypothesized that:

**Hypothesis** **1.** *Psychological capital will be positively associated with family satisfaction.*

**Hypothesis** **2.** *Occupational stressors will be negatively associated with family satisfaction.*

**Hypothesis** **3.** *Occupational stressors will moderate the association between psychological capital and family satisfaction. Specifically, when occupational stressors are higher, the positive association between psychological capital and family satisfaction will be weaker.*

## 2. Method

### 2.1. Study Design

A cross-sectional design was adopted in the current study. A survey comprised of scales measuring psychological capital, occupational stressors, family satisfaction and demographic information was administered online.

### 2.2. Participants and Procedures

The target population included employees from organizations in nine cities in the Greater Bay Area in mainland China (i.e., Guangzhou, Shenzhen, Zhuhai, Foshan, Huizhou, Dongguan, Zhongshan, Jiangmen and Zhaoqing). Past research on the interaction effect between psychological capital and occupational stressors found small effect sizes (*f*^2^ = 0.03 to 0.04) [43]. A power analysis was conducted using G * Power. It was revealed that our hypothesized moderation model required a minimum sample size of 208 to attain a statistical power of 0.80. The sample was obtained using a convenience sampling method during October and November 2020. A total of 787 eligible employees (367 males, 420 females) participated in an online survey. Participants were excluded if their response time was shorter than 2 s per item [44] or if the strings of their consistent responses were equal or greater than half the length of the survey [45]. Informed consent was obtained from all participants before the study. This study was approved by the Office of Research and Knowledge Transfer at Lingnan University, Hong Kong (reference number: EC-037/1920).

Among the participants in the current study, 21.86% (*n* = 172) of the participants were between the age of 25 and 29 years, 26.43% (*n* = 208) between 30 and 34 years, and 19.19% between 35 and 39 years (*n* = 151). Regarding their education level, 55.91% (*n* = 440) of them obtained a university degree. Regarding their marital status, 32.27% (*n* = 254) were single, 64.04% (*n* = 504) married or cohabited, 3.43% (*n* = 27) divorced or separated and 0.25% (*n* = 2) widowed. The mean organization tenure was 96.17 months (SD = 162.21). Regarding their position, 60.86% (*n* = 179) were non-managerial staff and 16.65% (*n* = 131) were front-line managers. Of the total participants, 60.61% (*n* = 477) worked for private institutions, 29.61% (*n* = 233) for public sector institutions, 2.54% (*n* = 20) for the government and 7.24% (*n* = 57) for state-owned enterprises. For their industry, 30.88% (*n* = 243) were in the manufacturing industry and 24.90% (*n* = 196) were in education. Finally, 37.10% (*n* = 292) of the participants earned a monthly salary of CNY 5001–CNY 9000.

### 2.3. Measures

Psychological capital: The Chinese version of Psychological Capital Questionnaire (PCQ-12; [25] was used to measure four components of psychological capital on a 6-Likert point scale (1 = strongly disagree, 6 = strongly agree): self-efficacy (three items), hope (four items), resiliency (three items) and optimism (two items). Cronbach’s αs of self-efficacy, hope, resiliency and optimism were 0.89, 0.86, 0.87 and 0.86, respectively, in our study. Cronbach’s α of the overall psychological capital questionnaire was 0.95.

Occupational stressors: As suggested by the Work Stress Management DIY Kit developed by the Occupational Safety and Health Council (OSHC) [46] and previous research [34], job insecurity, interpersonal conflicts, quantitative workload, organizational constraints and work–home interface were included as measures of occupational stressors. We adopted the Chinese measures of these occupational stressors used in past research [34]. A 6-point frequency-based response scale was used (1 = less than once a month or never, 6 = several times a day). The scale showed good reliability among the Hong Kong Chinese population [32]. Job insecurity was measured using three items from the Work Stress Management DIY Kit from the Occupational Safety and Health Council [46]. Quantitative workload was measured with five items adapted from Spector and Jex’s [47] Quantitative Workload Inventory. Organizational constraints were measured with 11 items taken from Spector and Jex’s [47] Organizational Constraints Scale. Interpersonal conflicts were measured with four items adapted from Spector and Jex’s [47] Interpersonal Conflict at Work Scale (ICAWS). The work–home interface was measured with three items from the OSHC [46]. The five scales showed good reliability in the current study (Cronbach’s αs ≥ 0.85). The Cronbach’s α of the overall occupational stressors was 0.96.

Family satisfaction: The three items measuring family satisfaction (Cronbach’s α = 0.95 in the current study) on a 7-Likert point scale (1 = strongly disagree, 7 = strongly agree) were taken from [48].

Demographic variables: Demographic information was measured, including age, gender, marital status, educational level, tenure in their current company, job position, current monthly salary, occupational group and the industry to which the employee belonged. The demographic variables were controlled in the analyses for an accurate estimate.

### 2.4. Data Analysis

Latent moderated structural equation modeling (LMS) with robust maximum likelihood estimation was carried out using Mplus to test our hypotheses. Four observations were removed due to missing data using the listwise deletion method. All items served as indicators for their respective first-order latent variables (family satisfaction, self-efficacy, resiliency, hope, optimism, job insecurity, interpersonal conflicts, quantitative workload, organizational constraints and work–home interface). Two second-order factors (psychological capital and occupational stressors) were specified, for that self-efficacy, resiliency, hope and optimism can be explained by psychological capital [25], and that job insecurity, interpersonal conflicts, quantitative workload, organizational constraints and the work–home interface can be explained by occupational stressors according to previous research [34]. The skewness and kurtosis of the indicators ranged from −0.73 to 1.79 and from −0.73 to 2.61, respectively, indicating that the indicators were normally distributed.

We firstly tested a structural equation model with the main effects of psychological capital and occupational stressors on family satisfaction. The model fit was evaluated with the comparative fit index (CFI), the Tucker–Lewis index (TLI), the root mean square error of approximation (RMSEA) and the standardized root mean square residual (SRMR). A well-fitting model can be indicated by a CFI ≥ 0.90, a TLI ≥ 0.90, an RMSEA < 0.05 and an SRMR < 0.08 [49,50,51]. An LMS model with the interaction of psychological capital and occupational stressors on family satisfaction was then tested. Because the aforementioned model fit indices are not produced for LMS models, a log-likelihood ratio test was carried out for model fit comparison [52,53].

## 3. Results

Descriptive statistics are shown in Table 1. The means of psychological capital (M = 4.04) and family satisfaction (M = 4.82) were above the scale mid-points, suggesting that the participants generally had high levels of psychological capital and family satisfaction, respectively. By contrast, the mean of occupational stressors (M = 2.20) was below the scale mid-point, suggesting that the participants generally had low levels of occupational stressors. Family satisfaction was positively correlated with psychological capital and negatively correlated with occupational stressors.

**Figure 1:**
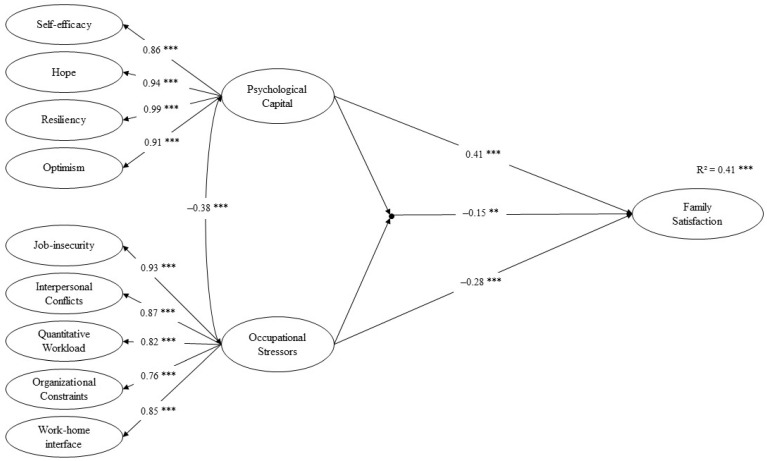
Model 1. Estimates are standardized. Observed variables and measurement errors are omitted for clarity. ** *p* < 0.01; *** *p* < 0.001.

The structural equation model that specified direct paths only from psychological capital and occupational stressors to family satisfaction (Model 0) displayed an adequate fit (χ^2^ (2078) = 3469.81, *p* <0.001, CFI = 0.93, TLI = 0.93, RMSEA = 0.03, 90% CI = [0.03, 0.03], SRMR = 0.07). The LMS model (Model 1, see Figure 1) with interaction of psychological capital and occupational stressors was then estimated. A log-likelihood ratio test revealed that Model 1 exhibited a better fit than Model 0 (log-likelihood difference value: D (df = 1) = 15.49, *p* < 0.001), indicating that adding the latent interaction term led to a significantly better model fit.

**Figure 2:**
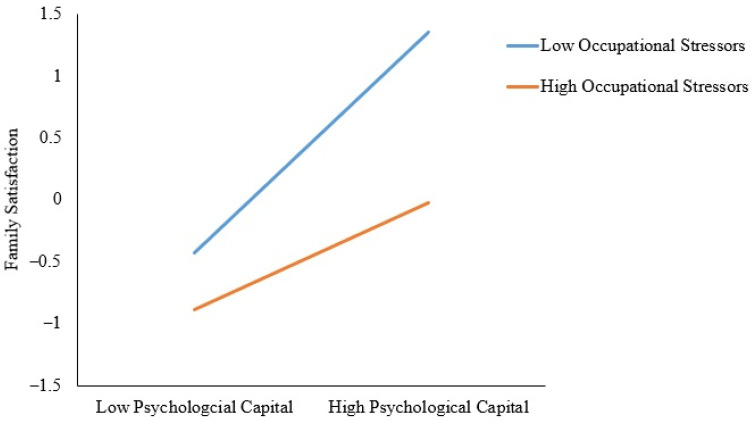
Interaction of psychological capital and occupational stressors on family satisfaction.

In the LMS model, the standardized factor loadings for the first-order latent variables ranged from 0.65 to 0.95, and the standardized factor loadings for the second-order latent variables ranged from 0.76 to 0.99, indicating good validity for the latent constructs. Significant main effects of psychological capital and occupational stressors were found. Family satisfaction was positively associated with psychological capital (*β* = 0.41, SE = 0.05, *p* < 0.001), and negatively associated with occupational stressors (*β* = −0.28, SE = 0.04, *p* < 0.001). A significant interaction was observed between psychological capital and occupational stressors on family satisfaction (*β* = −0.15, SE = 0.05, *p* = 0.004). Post-hoc analysis revealed that family satisfaction was positively associated with psychological capital (*β* = 0.56, SE = 0.06, *p* < 0.001) when the level of occupational stressors was low (M − 1SD). A weaker positive association between family satisfaction and psychological capital was found (*β* = 0.26, SE = 0.07, *p* < 0.001) when the level of occupational stressors was high (M + 1SD) (see Figure 2), suggesting that occupational stressors weakened the positive association between psychological capital and family satisfaction.

## 4. Discussion

The present study examined the association of family satisfaction with psychological capital and occupational stressors and the interaction of psychological capital and occupational stressors on family satisfaction. During the period of this study, the COVID-19 pandemic was stabilized in the Greater Bay Area in mainland China, and organizations resumed normal operations.

The results of structural equation modeling showed a valid higher-order structure of psychological capital, consisting of self-efficacy, optimism, hope and resiliency, consistent with Luthans et al. and Siu’s assumption [25]. Five major occupational stressors identified in previous research [34] were also validated to indicate occupational stressors as a second-order factor in the current sample.

Our results showed that family satisfaction was positively associated with psychological capital, supporting Hypothesis 1. The work–home enrichment model suggests that resources in the work domain can increase personal resources, which can be then utilized in the family domain [11,32]. Previous research has found that a higher level of psychological capital is associated with increased cognitive skills such as self-regulation [33] and improved work–family enrichment [54]. The observed association between psychological capital and family satisfaction may be explained by a work–home enrichment process in which resources generated by psychological capital (e.g., cognitive skills) in the work domain can be transferred to the family role.

We also found that family satisfaction was negatively associated with occupational stressors, which supported Hypothesis 2 and is in line with previous findings that stressors at the workplace were associated with diminished outcomes in other domains, such as quality of life and mental health [55,56]. According to the work–home conflict model [11], demands at the workplace exhaust resources, leading to a lackluster performance in the family domain. Empirical evidence has shown that occupational stressors have negative effects on outcomes in another domain (e.g., quality of life) through decreasing coping resources [57]. Following a similar process, occupational stressors may deplete resources, leading to unsatisfactory performance in the family domain and reduced family satisfaction.

Furthermore, our study demonstrated that occupational stressors weakened the positive association between psychological capital and family satisfaction, supporting Hypothesis 3. Psychological capital could mobilize personal resources [11,25], such as cognitive abilities [33]. Such resources are important for task completion at the workplace, which could generate positive effects, which could, in turn, be transferred into performance in the family domain. However, occupational stressors have a negative effect on such resources [58]. A weaker positive association between psychological capital and family satisfaction was observed when occupational stressors were higher, probably because occupational stressors diminished resources that could be mobilized by psychological capital, leading to a lower positive effect and fewer resources for the family domain, and hence lower family satisfaction.

### 4.1. Theoretical Contributions

To our knowledge, the present study is the first to test the interaction effect between psychological capital and occupational stressors on an outcome in the family domain. Previous research has mostly investigated the interaction effect of psychological capital and occupational stressors on outcomes in the work domain, such as creativity at work and interpersonal citizenship behavior [59,60]. Our findings suggest that there is a wider scope of domains that psychological capital and occupational stressors could interact to affect.

This work provides additional empirical evidence for the work–home resources model [11], the association between psychological capital and family satisfaction for the work–home enrichment process and the association between occupational stressors and family satisfaction for the work–home conflict process. Furthermore, the interaction effect between psychological capital and occupational stressors on family satisfaction provides clues to understand the work–home relationship when demands and resources are taken into account altogether. In the work–home resources model, conflict or enrichment arises as demands or resources in one domain diminish or improve outcomes in another domain through the loss or gain of resources, respectively. The present study has extended the work–home resources model by adding the interaction between demands and resources.

### 4.2. Practical Implications

Occupational stressors are ubiquitous and particularly high in Asia [61]. However, occupational stressors are harmful to employees. Apart from the negative consequences of occupational stressors found in previous research, the current study showed that individuals with higher occupational stressors were likely to have lower family satisfaction, which in turn may be associated with lower quality of life, mental health, life satisfaction, work–life balance and so forth [15,16,17]. Thus, it is important to reduce occupational stressors. Consistent with previous research [34], the current research used five major occupational stressors (job insecurity, interpersonal conflicts, quantitative workload, organizational constraints and work–home interface), providing hints for organizations to reduce employees’ occupational stressors as a primary stress intervention. For example, organizations may create a friendly environment, provide employees with adequate guidance, and give employees training to improve their coping skills.

Our study demonstrated that psychological capital was positively associated with family satisfaction, and this positive association remains, though weaker, even under high occupational stressors. As factors in the work domain and family domain have mutual effects on each other [11], family satisfaction may help to improve employees’ performance at work. The findings of the current study and previous research [62] together suggest that psychological capital is beneficial for employees and organizations. Organizations may deliver secondary stress interventions to employees to increase their psychological capital, drawing on [63] the psychological capital interventions approach. Alternatively, employees’ psychological capital may be promoted by organizational support [64], authentic leadership [65], external prestige [66] and so on.

### 4.3. Limitations and Future Directions

This study suffered from several limitations. The study used a convenience sampling method. In future research, it is suggested to use a more rigorous sampling method to obtain a more representative sample. Although we discussed that psychological capital may mobilize resources for task completion and occupational stressors may interfere with this potential process, the present study did not examine the resources that psychological capital may mobilize. Future research may test this potential process by including possible resources that psychological capital may marshal, to comprehensively understand the underlying mechanism of the interaction between psychological capital and occupational stressors.

Although structural equation modeling enabled the current study to observe that the endogenous variable (family satisfaction) could be predicted by the exogenous variables (psychological capital and occupational stressors), the cross-sectional design of this study fails to identify a causal relationship [67]. As factors in the work and family domain may have mutual effects on each other [11], a high level of family satisfaction might also promote psychological capital and reduce perceived stress at the workplace. Future studies could apply an experimental manipulation or a longitudinal design to further investigate the causal relationship among these variables.

This study did not control for the health status of the participants, and did not measure the influence of the COVID-19 pandemic on the relationship between psychological capital and family satisfaction. Moreover, apart from the occupational stressors measured, a perceived decrease in workplace security may be a potential moderator or mediator during the COVID-19 pandemic [68]. Further studies are suggested to include these variables.

In the current study, the association between psychological capital and family satisfaction was found among a Chinese population. Future research may examine whether cultural differences in the association between psychological and family satisfaction exist. Having grown up in a collectivistic society, Chinese individuals are more interdependent and tend to define themselves according to social roles in contrast to individuals in individualistic societies [69]. To fulfil their social roles, Chinese people may be more motivated to transfer the remaining resources to the family roles that psychological capital may mobilize for the work roles than individuals in individualistic societies. Thus, a weaker association between psychological capital and family satisfaction may be observed in individualistic societies.

## 5. Conclusions

To summarize, while occupational stressors were associated with decreased family satisfaction, psychological capital was positively associated with family satisfaction. The positive association between psychological capital and family satisfaction remained, though weaker when occupational stressors were high. These findings add empirical evidence for the work–home resources model and may suggest that it would be beneficial to enhance employees’ psychological capital and reduce their occupational stressors.

## Figures and Tables

**Table 1:** Means, standard variations, and correlation coefficients.

Construct	M	SD	1	2	3
1. Psychological capital	4.04	1.07			
2. Occupational stressors	2.20	0.95	−0.30 *		
3. Family satisfaction	4.82	1.66	0.50 *	−0.40 *	
4. Tenure (in months)	96.17	162.21	0.12 *	−0.10 *	0.11 *
5. Male	0.47	0.50	−0.10 *	0.10 *	−0.10 *
6. Bachelor’s degree or above	0.56	0.50	−0.03	0.08 *	−0.04
7. Age 35 or above	0.43	0.50	0.18 *	−0.25 *	0.16 *
8. Ever married	0.68	0.47	0.10 *	−0.10 *	0.10 *
9. Managerial staff	0.39	0.49	0.03	0.27 *	−0.12 *
10. Public sector or private institution	0.30	0.46	0.10 *	−0.10 *	0.11 *
11. Manufacturing industry	0.31	0.46	0.16 *	−0.24 *	0.17 *

Note. Male, bachelor’s degree or above, age 35 or above, ever married, managerial staff, public sector or private institution and manufacturing industry are dummy coded. The reference categories for the dummy variables are female, below bachelor’s degree, age below 35, never married, non-managerial staff, government or state-own enterprise and other industries, respectively. * *p* < 0.05.
